# Structural insight into the type-specific epitope of porcine circovirus type 3

**DOI:** 10.1042/BSR20201109

**Published:** 2020-06-15

**Authors:** Mingfang Bi, Xiangdong Li, Weifeng Zhai, Bo Yin, Kegong Tian, Xiaobing Mo

**Affiliations:** 1 College of Veterinary Medicine, Jilin University, Changchun 130062, China; 2 Department of Biological Sciences and Centre for Bioimaging Sciences, National University of Singapore, 117543, Singapore; 3 National Research Center for Veterinary Medicine, Road Cuiwei, High-Tech District, Luoyang 471003, China; 4 College of Animal Science and Veterinary Medicine, Henan Agricultural University, Zhengzhou, 450002, China

**Keywords:** capsid protein, cryo-EM structure, Porcine circovirus type 3 (PCV3), prophylactic vaccine, type-specific epitope, virus-like-particle (VLP)

## Abstract

The recently identified pathogenic Porcine circovirus type 3 (PCV3) may threaten to reduce the pig population dramatically worldwide. In our previous study, a PCV3-specific monoclonal antibody (mAb-1H11) was successfully applied in immune-histochemistry staining and ELISA, which specifically recognize PCV3 capsid protein in PCV3-positive pig tissues. In the present study, we expressed and purified the soluble sole capsid protein of PCV3. The purified capsid protein was capable of self-assembly into virus-like-particles (VLPs), which is validated by transmission electron microscopy and dynamic light scattering assays. Moreover, the epitope of mAb-1H11 was identified in the CD-loop region (a.a. 72-79) on the VLP surface, which is confirmed by PCV2-PCV3 epitope swapping assay. For the first time, we determined the cryo-EM structure of PCV3-VLP at 8.5 Å resolution that reveals the detailed structural information of PCV3-VLP. In our cryo-EM structure, PCV3-VLP is composed of 60 capsid protein subunits assembled with *T* = 1 icosahedral symmetry. Consistent to our bio-dot Western blot assay, the structural comparison between PCV3 and PCV2 revealed significant structural differences in the surface-exposed loops, including the CD-loop (a.a. 72-79) and the EF-loop (a.a. 109-131). Our work provides a structural framework for engineering future PCV3 vaccine and diagnosis kits development.

## Introduction

Porcine Circovirus (PCV) belongs to the Circovirus genus in the Ciroviridae family, which is considered as one of the most economically disastrous viral pathogens worldwide. For a long time, there were only two types of PCV: PCV1 and PCV2. PCV1 is believed as non-pathogenic for pigs, whereas PCV2 is considered as the major pathogenic agent leading to the porcine associated disease (PCVAD). As the new-emerging porcine circovirus, porcine circovirus type 3 (PCV3) and PCV4 were first reported in the United States and in China, respectively [1,2]. Since 2016, PCV3 has been detected in farms across multiple countries, including Poland, South Korea, Italy, Brazil and Mainland China [3–8]. PCV3 has been frequently reported to be associated with porcine dermatitis and nephropathy syndrome, congenital tremors, reproductive failure, and multi-systemic inflammation [8–12]. Recently, PCV3 infection with a PCV3 DNA clone can lead to Porcine Dermatitis and Nephropathy Syndrome in Piglets [13].

PCV3 infection cases usually have severe clinical symptoms and have been detected in many swine farms worldwide [5,9,14–17]. Although PCV3 has been found to be associated with similar syndrome to PCV2, PCV3 is a new porcine circovirus that may have originate from a bat-associated circovirus approximately via by inter-species transmission [3]. However, low genome sequence identities (∼27%) between capsid protein sequences of PCV3 and other genotypes of PCV limits the effectiveness of PCV2 vaccine; there is no cross-protection of PCV3 infection with PCV2 vaccine observed nor do anti-sera of PCV3 cross-react with PCV1 and PCV2 samples [18]. As reported, the engineered PCV2 vaccine, developed from the self-assembled VLP from PCV2 capsid protein, shows superior prophylactic efficacy compared with traditional vaccines created from inactivated PCV2 viruses [19–21]. Therefore, it is urgent to investigate the pathogenesis of PCV3 both at molecular and structural level, which can provide useful ideas for the design and development of PCV3 diagnosis kits and vaccines.

As one of the smallest spherical viruses [22], PCV has a single-stranded, circular DNA genome (PCV1: 1760 bp; PCV2: 1767–1777 bp), which contains 11 major open reading frames (ORFs), ORF1, ORF2 and et al. ORF1 encodes the replicate-associate protein, and ORF2 encodes the capsid proteins. Notably, the genome of PCV3 is a little bit longer (∼2000 nucleotides in length) than those in PCV type 1 and 2, and encodes an additional ORF (ORF3) with unknown function [1,4]. The PCV3 sole capsid protein (214 amino acids), encoded by the ORF 2, is capable of self-assembly into virus-like particles (VLP) in a *T* = 1 icosahedral symmetry, morphologically similar to that of native PCV2 virions [23].

There are several known crystal structures and cryo-EM structures of PCV2 capsid protein: 9.6 Å cryo-EM structure of full-length PCV2-VLP showed the icosahedral symmetry of the virus, which is assembled from 60 subunits capsid protein; the 2.3 Å crystal structure of *in vitro* assembled PCV2-VLP with N-terminus truncated (3R0R) revealed the structural details of the surface loops, which may serve as the immunodominant epitopes that elicit neutralization antibodies [24]. The 2.9 Å cryo-EM structure of PCV2-VLP under close-packed condition (EMD-6555) also revealed an N-terminus truncated PCV2-VLP structure [25]. In our previous study, the 2.8 Å crystal structure of PCV2-VLP (PDB: 5JZU) derived from Nuclear Localization Signal (NLS) truncated capsid protein showed that NLS-truncated PCV2 capsid protein can form unstable and easily disassembled VLPs, and the 4.1 Å cryo-EM structure of full-length PCV2-VLP (EMD-6746) showed for the first time, the interaction between ^15^PRSHLGQILRRRP^27^ (α-helix) and ^33^RHRYRWRRKN^42^ (NLS-B) in NLS structure stabilizes the assembled PCV2 VLP in solution [26]. The 2.8 Å cryo-EM structure of PCV2b-VLP (EMD-8969) revealed an asymmetric distribution of heparin on the surface of the virus capsid of PCV2 [27]. The 3.3 Å PCV2d-VLPs (EMD-20113) pointed out the capsid protein N-terminus responds to the packaged nucleic acid [28].

So far, the structure of PVC3 capsid protein has not been reported, although the detailed structural characterization of how PCV3-VLP assemble *in vitro* can provide clues on the design and development of effective PCV3 vaccine.

In our previous study, we identified a PCV3-specific monoclonal antibody, 1H11, which can distinguish PCV3 from other PCV genotypes specifically and accurately [29]. In the present study, we expressed a soluble form of PCV3 capsid protein with SUMO-tag using *Escherichia coli* expression system and successfully obtained *in vitro* assembled PCV3-VLPs. Based on the bio-dot Western blot detection of affinities between mAb-1H1 and chimeric PCV3 capsid proteins with swapped loops from PCV2, we showed that the epitope against mAb-1H1 is located in the CD-loop on the surface of the PCV3. In addition, we determined the cryo-EM structure of PCV3-VLP to 8.5Å resolution, showing unique structural features of N-terminus and the CD-loop of PCV3 capsid protein. Application of indirect ELISA experiments with PCV3 capsid protein showed that PCV3 capsid protein can be used to diagnose PCV3 infected pigs. Taken together, our results provide structural information for the development of PCV3 prophylactic vaccine and diagnosis kits.

## Materials and methods

### Cloning, protein expression and purification of PCV3 capsid protein

Full-length codon optimized synthetic open reading frame 2 (ORF2, Accession number: KX458235.1) of porcine circovirus type 3 (PCV3) was amplified by DNA polymerase and cloned into a pET28a-SUMO vector. The constructed expression plasmid (pET28a-SUMO-ORF2) was transformed into *E. coli* BL21 (DE3) for fusion-expression. The cells were grown in LB media supplemented with Kanamycin antibiotics to an OD_600_ value reaching 0.6; a final concentration of 0.4 mM IPTG was added to induce the recombinant protein expression. Cells were cultured overnight and harvested by centrifugation. The pellets were resuspended in lysis buffer containing 20 mM Tris (pH 7.4), 300 mM NaCl, 0.5% Triton X-100, 1 mM DTT and 2 mM EDTA and disrupted by homogenizer for four times. After ultracentrifugation at 20,000 rpm for 1 h, the supernatant was collected and purified with a combination of Ni-NTA purification, cleavage of SUMO-tag by ULP1 protease and followed by dialysis and Ni-NTA flowthough purification in a buffer containing 20 mM Tris (pH 7.4), 500 mM NaCl. Finally, the capsid protein was polished by size-exclusion chromatography in a buffer containing 20 mM Tris (pH 7.4), 500 mM NaCl, 2 mM DTT and 0.5 mM EDTA. The purified PCV3 capsid protein was dialyzed against a buffer containing 20 mM phosphate buffer and 500 mM NaCl for *in vitro* VLP assembly.

### Dynamic light scattering (DLS) assay

The dynamic light scattering (DLS) measurements were performed at room temperature on a DynaPro (protein solution) DLS instrument. Before measuring, all protein samples and control buffers were filtered through a 0.22-μm filter to avoid any dust and unwanted aggregates, and then degassed on a thermal vacuum followed by ultracentrifuge for at least 15 min. The cuvette was successively rinsed with Milli-Q water, 100% methanol, and filtered water again for several times to clean and remove dust. Before sample measurement, the sample buffer was loaded into the cuvette and measured as a blank control. For each sample, at least 20 acquisitions were collected for data analysis.

### Negative stain electron microscopy (TEM) of PCV3-VLP

About 8 μl of purified full-length PCV3-VLP specimen (1 mg/ml) was applied to carbon-coated copper grids, which were pretreated by glow discharge. The extra sample was removed by filter papers and the sample-loaded grids were treated with 5% phosphate-tungstic acid (PTA), and then dried. Images were taken on an FEI Tecnai-12 electron microscope operated at 120 kV, with a magnification of 67,000×.

### Cryo-EM data collection and 3D structure reconstruction of PCV3-VLP

About 5 μl of purified full-length PCV3-VLP (1 mg/ml) specimen was loaded onto a Quatifoil 2/1 grid, blotted with filter papers (two times, 2 s per blotting) and rapidly plunged into liquid ethane, pre-cooled by liquid nitrogen. Cryo-EM images were taken from the frozen grids in a Titan Krios electron microscope operated at 300 kV, with a magnification of 67,000× and a pixel size of 1.69 Å/pixel. Measured defocus values of these images range from -1.5 μm to -4 μm. Approximately ∼10,500 individual particles for full-length PCV3-VLP were excised from micrographs by boxing and the contrast transfer function (CTF) correction for each micrograph was determined by EMAN2 (blake.bcm.tmc.edu/EMAN2), based on incoherently averaged Fourier transforms of each image. The particle sets were classified and averaged. Only the good average classes indicate the hexagon characters were subjected to make initial models by the application of icosahedral symmetry. About 4985 particles were used for 3D reconstruction using EMAN2 software. The final effective resolution of the map is ∼8.5 Å, when 0.143 Fourier shell correlation criteria are used. The densities of PCV3-VLP were extracted from the density map and visualized using UCSF Chimera software (www.cgl.ucsf.edu/chimera).

### Real space refinement of PCV3 model against PCV3 cryo-EM map

The medium resolution modeled structure was further refined for 20 cycles against the cryo-EM structure of PCV3-VLP at 8.5Å using the ‘real space refinement’ module in Phenix [30], and the statistics were listed in Table 1.

**Table 1:** Statistics table of data collection, 3D reconstruction and real space refinement of PCV3-VLP model against cryo-EM map of PCV3-VLP

**Data collection**	
Microscope	FEI Titan Krios
Voltage (kV)	300
Dose (e^−^/Å^2^)	37
Detector	Falcon II
Pixel size (Å)	1.69
Defocus range (μm)	−1.5 to −4.0
**Reconstruction (EMAN2)**	
Micrographs (Initial/Final)	101/77
Particle number (Initial)	10,500
Particle number (Final)	4,985
Symmetry	Icosahedral
Box size (pixels)	200
Sharpening B-factor (Å^2^)	−350
Final resolution (Å)	8.5
EMDB accession code	EMD-6935
**Model Refinement (PHENIX)**	
Cross correlation (Whole Volume)	0.750
Cross correlation (Masked)	0.740
**Ramachandran Plot**	
Outliers	0.00%
Allowed	11.60%
Favored	88.40%

### Indirect enzyme-linked immunosorbent assay

About 96-well plates were coated with recombinant PCV3 capsid protein in carbonate-bicarbonate buffer (pH-9.6) and incubated at 37°C for 20 min. After washing three times with PBST (PBS containing 0.05% Tween-20), the plate was blocked with 5% BSA for 2 h at 37°C. About 100 μl pre-selected serum samples were added and incubated for 30 min at 37°C. The sample wells were then washed three times with PBST, followed by incubation for 15 min at 37°C with HRP-labeled goat anti-pig IgG (Sigma-Aldrich, Shanghai, China). After washing five times with PBST, 100 μl of substrate was added and incubated for 15 min at room temperature. At last, the reaction was stopped using 100 μl of 2M H_2_SO_4_ and the optical density was recorded at λ_450_ (wavelength: 450 nm) using an ELISA plate reader.

## Results

### PCV3-VLP assembly from PCV3 capsid protein

**Figure 1:**
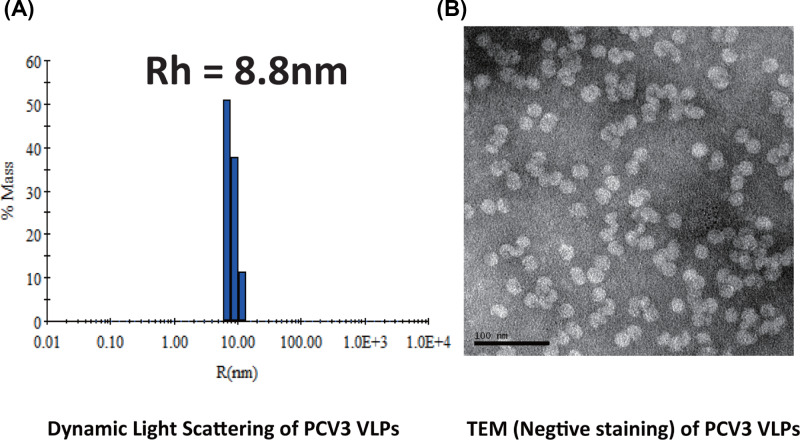
Full-length PCV3 capsid proteins assemble into VLPs (**A**) Dynamic light scattering measurement of PCV3 VLPs. The enlarged area showed that the average hydrodynamic radius of PCV3 VLP is ∼8.80 nm and the VLP assembly rate is ∼95%. (**B**) Transmission electron microscopy of PCV3 VLPs. The scale bar is 100 nm long and EM result indicated that the PCV3 capsid proteins are assembled into VLPs.

In the present study, the full-length and soluble PCV3 capsid protein was expressed with N-terminal SUMO-tag in *E. coli.* The PCV3 capsid protein was purified following the routine protocols described in a previous study [23]. Briefly, a multi-step purification process was applied including affinity chromatography (Ni^2+^-NTA) purification, SUMO cleavage by ulp1 (nickel-chelating), Ni^2+^-NTA to remove SUMO-tag and followed by size-exclusion chromatography purification. Next, the purified PCV3 capsid protein was further dialyzed against the buffer containing 20 mM phosphate buffer (pH 6.5) and 500 mM NaCl for *in vitro* VLP assembly. The completeness and correctness of the PCV3-VLP assembly were validated by dynamic light scattering (DLS) assay and transmission electron microscopy (TEM). As expected, the DLS assay showed that ∼95% of PCV3 capsid proteins self-assembled into VLPs in the assembly buffer, with an average radius of ∼ 8.8 nm (Figure 1A). Consistently, the radius of the majority of PCV3 particles is 7.5–10 nm under transmission electron microscopy imaging (Figure 1B).

### Cryo-EM structure of PCV3-VLP

In literature, the widely used recombinant VLP-based prophylactic vaccine offers one of the best means to stimulate the immune system by facilitating antigen presentation, uptake and recognition. Such strong response is attributed to the structural similarity between VLPs and its pathogenic virus [31].

**Figure 2:**
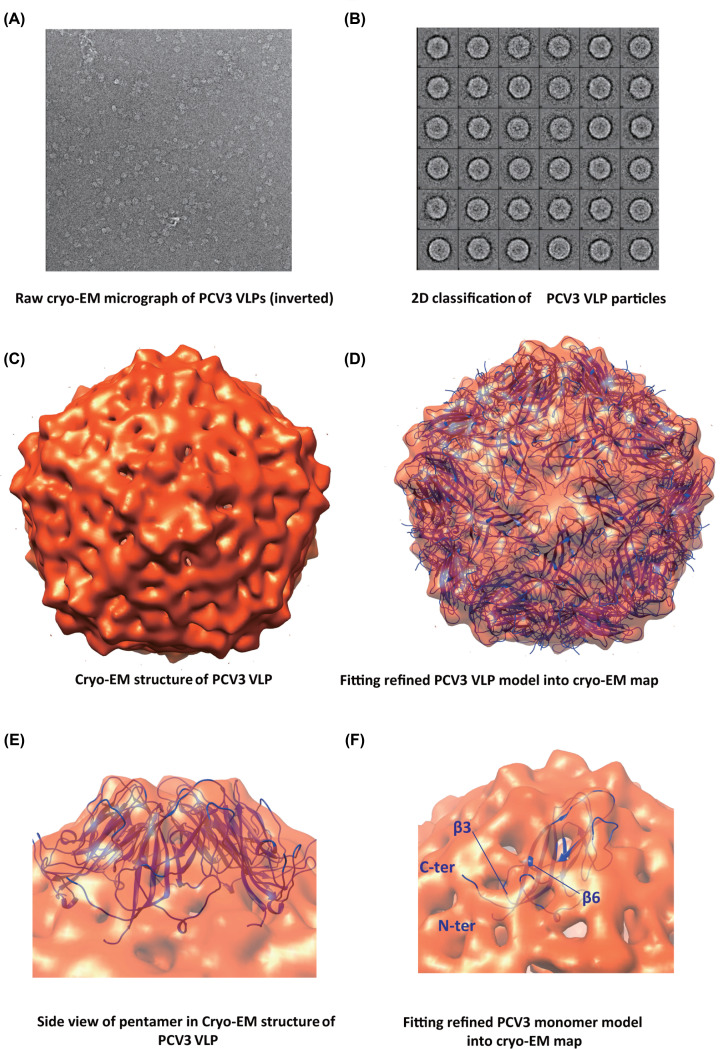
Cryo-electron microscopy study of PCV3-VLP (**A**) Raw cryo-EM micrograph of PCV3-VLP (inverted). (**B**) 2D classification of PCV3-VLP particles. Most classes showed the structural characteristics of icosahedral particles. (**C**) Cryo-EM structure of PCV3-VLP at 8.5 Å resolution, the density maps are colored in red. (**D**) Refine PCV3-VLP model fitting into the cryo-EM map (EMD-6935) of PCV3-VLP, in which PCV3 model structure is shown in blue ribbon mode. (**E**) Side view of pentamer in the cryo-EM map (EMD-6935) of PCV3-VLP, in which PCV3 pentamer model structure is shown in blue ribbon mode. (**F**) Refine PCV3-VLP monomer fitting into the cryo-EM map (EMD-6935) of PCV3-VLP, in which PCV3 model structure is shown in blue ribbon mode.

To investigate the structural features of the PCV3 capsid protein and provide the structural basis for PCV3 vaccine development, we have collected 101 micrographs under low radiation dose conditions. Next, contrast transfer function (CTF) correction for each micrograph was applied to each image based on incoherently averaged Fourier transformation by EMAN2 (blake.bcm.tmc.edu/EMAN2). Approximately 10,500 individual particles were excised from the 77 best micrographs. The particle sets were classified into 60 classes and the selected particles were averaged. The averaged classes with good and apparent icosahedral projection shapes were used to make initial models by the application of icosahedral symmetry. At last, 4985 best particles were used for final 3D refinement. The final resolution of this refined cryo-EM structure of PCV3-VLP was determined to ∼8.5Å resolution (Figure 2A–C), in which the local resolution distribution was assessed by FSC curve and Resmap/Chimera [32] (Supplementary Figure 1A,B). The statistics table of the data collection and 3D reconstruction are listed in Table 1.

The reconstructed EM map shows a significant resolution variation ranging from 8.5 to 9.5 Å at the outer surface and inner concave regions to 8.0–8.5 Å at the β-strand barrel core regions (Supplementary Figure S1C,D). The relatively low resolutions of the outer surface and inner N-terminal regions of PCV3-VLP suggest these regions in PCV3 capsid are flexible (Supplementary Figure S1C). In our cryo-EM structure of PCV3-VLP, densities of most of its secondary structures can be traced with the visual examination (Figure 2D–F). The densities of the surface-exposed loops in the loop-BC and loop-CD region are lower than other parts, possibly due to increased conformational flexibility in these exposed loops.

### Structural comparison of PCV3 with PCV2

Similar to PCV2 VLP structures [24–28,33], our cryo-EM structure of PCV3-VLP clearly shows icosahedral symmetry with 60 capsid protein subunits assembled with *T* = 1 icosahedral symmetry, with 2-, 3- and 5-fold axes clearly observed (Supplementary Figure S2). To reveal the unique structural characters of the PCV3-VLP, model building against the medium resolution cryo-EM structure of PCV3 was performed using PHENIX software (‘map to model’ module in PHENIX, version 1.17rc1-3605), followed by 20 rounds of real space refinement against our cryo-EM map (EMD-6935). The statistics for the PCV3-VLP model refinement against our cryo-EM density map are also listed in Table 1.

**Figure 3:**
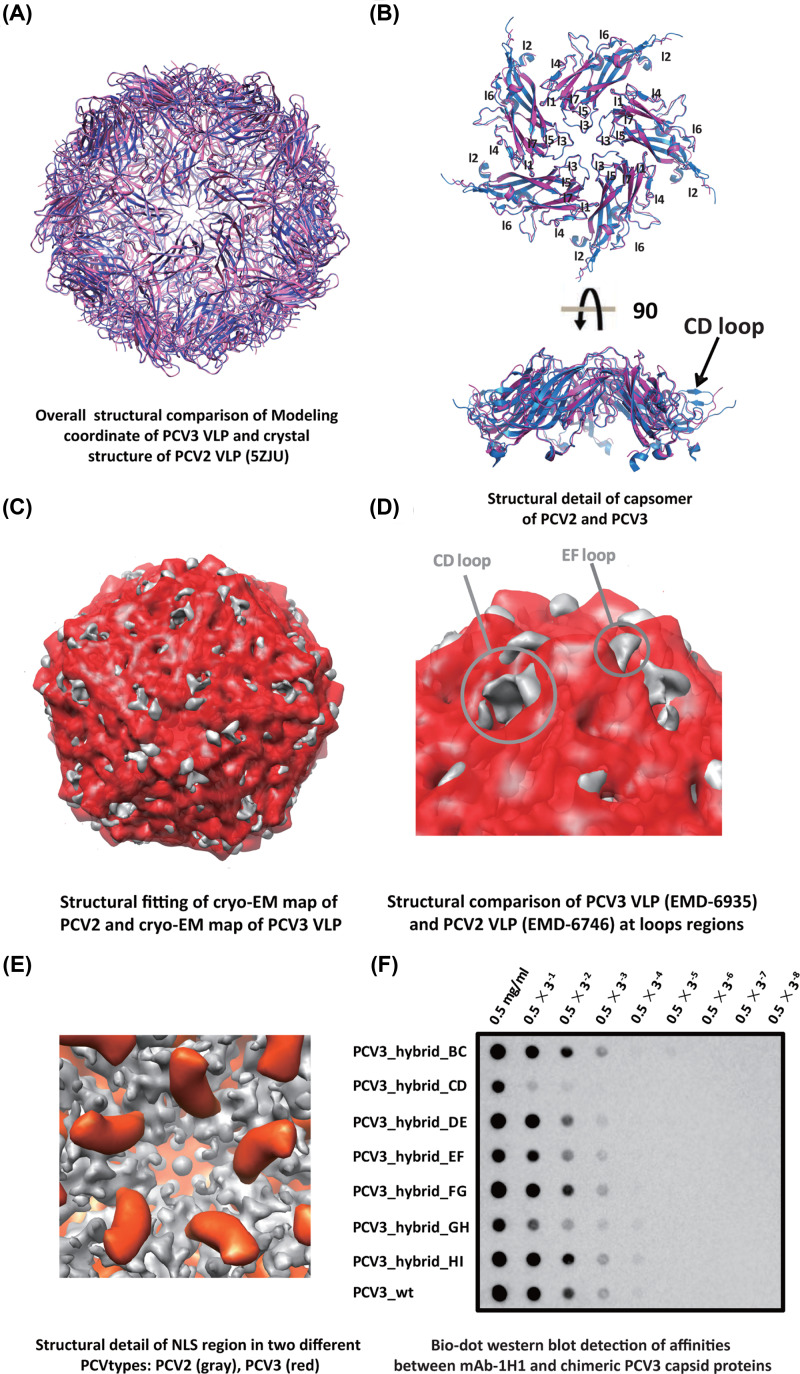
PCV3 type-specific epitope mapping and Structural comparison between PCV2 and PCV3 (**A**) Overall structural comparisons of PCV3-VLP model and crystal structure of PCV2 VLP (5ZJU), in which the PCV2 VLP and PCV3-VLP are shown in pink and blue ribbon mode, respectively. (**B**) Structural comparison of capsomers of PCV2 and PCV3 capsid proteins, viewed from the side and top orientations, respectively. Most of the seven flexible surface loops of PCV capsomer are surface exposed. (**C**) Structural fitting of PCV2 and PCV3 VLPs. PCV3 density maps are colored in red, whereas PCV2 density maps are colored in gray. (**D**) Structural comparison of PCV2 VLP and PCV3 VLP at loop regions. Detailed structural comparison of CD-loops and EF-loops between PCV2 and PCV3 VLPs. PCV3 VLP density maps are colored in red, whereas PCV2 VLP density maps are colored in gray. (**E**) Structural details of the N-terminal fragments including the NLS regions of PCV2 cryo-EM structure (EMD-6746) and PCV3 cryo-EM structure (EMD-6935). PCV3 density maps are colored in red, whereas PCV2 density maps are colored in gray. (**F**) Bio-dot Western blot detection of affinities between PCV3 type-specific mAb and chimeric PCV2 capsid proteins with swapped loops from PCV2. Swapping of PCV2 capsid protein CD-loop with the corresponding PCV1 capsid protein loop disrupts the binding between PCV2 capsid protein and mAb-1H1.

This refined built model of PCV3-VLP showed significant structural conservation among different types of PCVs in general (Figure 3A and Supplementary Figure S3A,B). Notably, significant structural differences between PCV2 and PCV3-VLP are clearly observed among loops primarily located on the outside surface of the PCV3-VLP capsid [26], such as CD-loop (a.a. 72-79) and EF-loop (a.a. 109-131) (Figure 3B–D). Compared with the PCV2 CD-loop, the PCV3 CD-loop showed lower densities in our cryo-EM structure, suggesting that either the conformation of PCV3-CD-loop is significantly different from that of PCV2-CD-loop or that the structure of PCV3-CD-loop is more flexible (Figure 3B). Consistently, sequence alignment and structural comparison strongly suggested that PCV3 CD-loop is shorter than PCV2 CD-loop (8 residues vs. 16 residues) (Supplementary Table S1). Residues located on PCV3 CD-loop are less conserved than the residues in the core β-strands regions and other loop regions (Supplementary Figure S4), as our cryo-EM structure of PCV3-VLP (EMD-6935) shows density at its N-terminal fragment (Figure 3E), which is considered to be highly flexible in literature [24,25] but has been observed at our recent determined cryo-EM structure of PCV2-VLP (EMD-6746) [26].

The conformation of N-terminal part of PCV3 capsid protein revealed from our cryo-EM density (EMD-6935) is different from that of PCV2 capsid protein revealed from the cryo-EM density, suggesting the structural flexibility and multifunctional roles of N-terminal fragment of PCV capsid protein (Figure 3E). In the cryo-EM structure of PCV2 (EMD-6746) [19], the interaction between the Arginine-rich residues (^15^PRSHLGQILRRRP^27^, α-helix) in NLS of one capsid protein and the adjacent NLS-B fragment from another capsid protein which stabilizes the VLP formation. In our cryo-EM structure of PCV3 (EMD-6935), structural flexible NLS fragments have trajectory at different locations/orientations that can be seen on the cryo-EM density at current contour level.

### Mapping PCV3 type-specific epitope

There are four genotypes of porcine circovirus reported so far, such as PCV1, PCV2, PCV3 and PCV4 [2]. The types 2 and 3 of PCV are considered threatening pathogens, causing PMWS in piglets, whereas PCV1 is not. Moreover, PCV is reported to constantly evolve under vaccination pressure, which result in emergence of new PCV strains and genotypes through genome mutation/recombination or genotypic shift: there are two globally genotypic shifts from PCV2a towards PCV2b occurred around 2003; genotype prevalence shift from the predominant PCV2b toward PCV2d [26,34–36]. Hence, it is critical to identify the critical epitope on the PCV3 for the development of next-generation PCV3 diagnostic kits to monitor the co-spreading of different PCV strains and genotypes.

Based on sequence alignment, the majority of the deviated sequences of the three PCV genotypes (PCV1, PCV2 and PCV3) are located within the surface-exposed loop regions (Supplementary Figure S4). It was reported that the capsid sequence changes among the analyzed circovirus isolates do not yield major structural changes in the overall β-strands core region and capsid viral assembly but instead on the epitope regions [37]. Therefore, we speculate that the PCV3 type-specific monoclonal antibody, 1H1, probably have the ability to distinguish PCV3 from other genotypes by recognizing one of the surface epitopes on the loop regions. Notably, the PCV2-specific epitope was identified on the EF-loop on the surface of PCV2 in our previous study using the same strategy [26].

To this end, we prepared chimeric PCV3 capsid proteins by replacing the capsid protein surface loop sequence with corresponding PCV2 capsid protein sequence, and performed systematic affinity screening between a PCV3 type-specific mAb, named 1H1, and chimeric wild-type/chimeric PCV3 capsid proteins. Notably, we have made seven chimeric PCV3 capsid proteins by replacing the loop sequences at the following loop regions one by one: BC-loop (residues 58–66), CD-loop (residues 79–94), DE-loop (residues 108–116), EF-loop (residues 124–146), FG-loop (residues 153–156), GH-loop (residues 162–193) and HI-loop (residues 204–208) (Supplementary Table S1). Bio-dot Western assays were performed to test the binding affinities between the mAb-1H1 and the purified wild-type/chimeric capsid proteins. Notably, the replacement of PCV3 CD-loop sequence with corresponding PCV2 CD-loop sequence compromised the binding of mAb-1H1 to PCV3-CD-loop-exchanged capsid protein (Figure 3F), and the results indicated that the mAb-1H1 may recognize one epitope on the PCV3 CD-loop (residues 128–143). By comparison, replacement of other PCV3 loops had an insignificant impact on mAb-1H1 binding with purified chimeric PCV3 capsid proteins. Moreover, sequence comparison also suggested significant sequence variations on the CD-loop on the capsid protein between PCV2 and PCV3 were observed (Supplementary Figure S4).

## Discussion

### Structural and sequence comparisons of PCV capsid proteins

Porcine circovirus causes post-weaning multi-systemic wasting disease (PMWS) and other associated diseases, leading to severe economic loss in the swine industry worldwide [9,38]. In PCVs, the sole capsid protein encoded by ORF2 serves as the immune-dominant antigen stimulating host immune response. In the present study, we developed a robust protocol to express and purify PCV3 capsid protein using *E. coli* expression system and have obtained *in vitro* assembled PCV3-VLPs. PCV3-VLPs can be used as a prophylactic PCV3 vaccine candidate. In addition, we have determined the cryo-EM structure of PCV3-VLP at the resolution of 8.5 Å. Our cryo-EM structure of PCV3-VLP, supplemented by modeled coordinate and read-space refinement, revealed the structural details of PCV3 capsid protein including the low density of the N-terminal fragment and the flexible loop regions. This critical information can be used for future PCV prophylactic vaccine development.

Structural comparisons and sequence alignment of PCV capsid proteins showed that PCV capsid proteins contain two highly conserved regions, β3 and β6, and two significant different loops, CD-loop and EF-loop (Figure 3B–D). We speculate that the highly conserved β3, and β6 may play significant roles in PCV particle assembly/stabilization, host entrance or PCV replication. Consistently, both β3 and β6 are located in the inner core region within the assembled VLP (Figure 2F), whereas NLS is believed to participate in PCV replication in host or particle stabilization [39]. Notably, our cryo-EM structure of PCV3-VLP (EMD-6935) clearly shows the density for the N-terminal NLS region. Interestingly, the location/orientation of the density of NLS fragment in PCV3 capsid is different from that of PCV2b-VLPs and PCV2d-VLPs structure [26] (Figure 3E), suggesting the relationship between structural flexibility and multi-functions of this conserved fragment among PCV family members (Figure 3E). The amino acids 36-42 in the N-terminus of both PCV2b and PCV2d molecules are located near the icosahedral 3-fold axes of symmetry [26,28]. PCV2 NLS-A fragment is reported to be used as a versatile vehicle to transport macromolecules across cellular bio-membranes, or as a cell-penetrating peptide (CPP) to facilitate viral entry into the cell by enhancing intracellular delivery of genome DNA during viral infection [40,41]. NLS-A can reach outside the virus’ surface and interact with host receptor proteins with its patch of basic-charged residues. Although PCV3 capsid protein shares 35–40% amino acids identities with PCV2 capsid protein, PCV3 is distinct in Phylogenetic relationship with PCV2. There is no cross-reaction reported between the PCV3 antiserum and PCV2 VLPs [18]. Consistently, structural comparison of PCV3-VLP and PCV2 VLP demonstrates the significant structural variations among the surface-exposed loops, such as BC-loop (a.a. 51-59), CD-loop (a.a 72-79) and EF-loop (a.a. 109-131). Amongst them, the shorter CD-loop of PCV3 capsid protein contains much lower densities and significantly different structural conformations as compared with those of PCV2 capsid protein. Our previous reports show that PCV3-VLPs could produce PCV3-specific monoclonal antibodies in the host [29].

Taken together, we have reported the strategies to produce high-quality PCV3-VLPs using *E. coli* expression system and determined the cryo-EM structure of PCV3-VLP at ∼8.5Å for the first time. Our results show that PCV3-VLP displays a typical *T* = 1 icosahedral symmetry, which contains type-specific surface epitopes discriminating for different genotypes of PCV (Supplementary Figure S5). Our results provide new structural insights on PCV3 morphology which is crucial in the design of robust VLP-based prophylactic PCV3 vaccines and diagnostic kits.

## Highlights

1. Soluble full-length PCV3 capsid proteins are expressed in *E. coli* system and undergo self-assembly (*in vitro*) into virus-like-particles (VLPs).
2. The PCV3-specific epitope was identified on the surface of the CD-loop region of PCV3-VLPs
3. For the first time, we had determined the cryo-EM structure of *E. coli* expressed and *in vitro* assembled full-length PCV3-VLP, which was refined to 8.5Å resolution.
4. Significant structural variations on the CD-loop on the capsid protein between PCV2 and PCV3 were observed.

## Supplementary Material

Supplementary Figures S1-S5 and Tables S1

## Abbreviations

CPP: cell-penetrating peptide
CTF: contrast transfer function
ORF: open reading frame
PCV3: Porcine circovirus type 3
VLP: virus-like-particle
