# Supplementary material for: Structural insight into the type-specific epitope of porcine circovirus type 3
Source: Biosci Rep. 2020 Jun 15;40(6):BSR20201109. doi: 10.1042/BSR20201109 (PMC7295619; doi:10.1042/BSR20201109)
Supplement: Supplementary Figures S1-S5 and Tables S1 [file BSR-2020-1109_supp.pdf]

Fig S1. Bi et al.

A

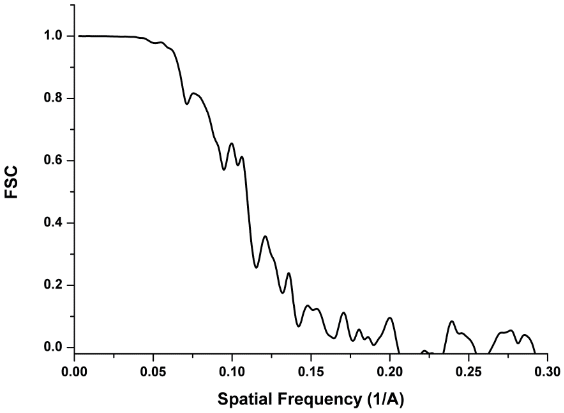

Gold Standard FSC curve of cryo-EM structure of PCV3 VLP

B

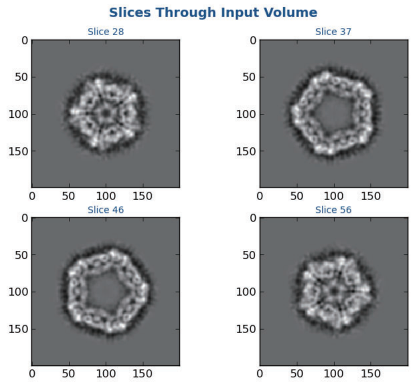

Slices Through Input Volume

C

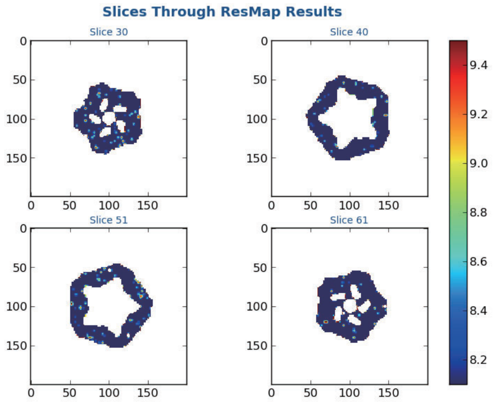

Slices Through ResMap Results

D

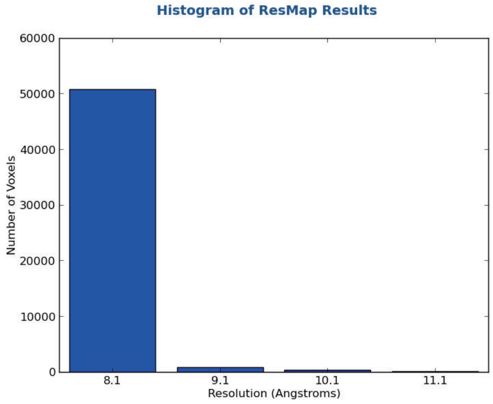

Histogram of ResMap Results

Fig S1. Local resolution assessment of 3D reconstructed cryo-EM structure of PCV3 VLPs. (A) The Fourier Shell Correlation curve of resolution estimation of the data set used for our 3D reconstruction. (B) Slices through input volume. (C) Slices through ResMap results. (D) Histogram of ResMap results.

Fig S2. Bi et al.

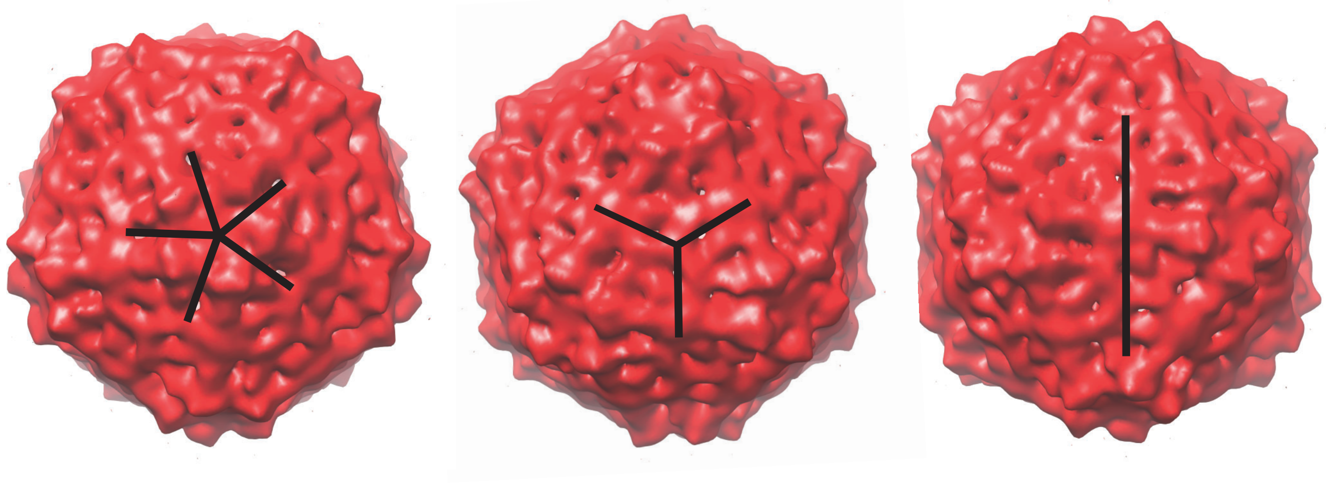

5-fold axis 3-fold axis 2-fold axis

Icosahedral symmetry of cryo-EM structure of PCV3 VLP

Fig S2. The 5-, 3-, and 2-fold axis in the cryo-EM structure of PCV3

Fig S3. Bi et al.

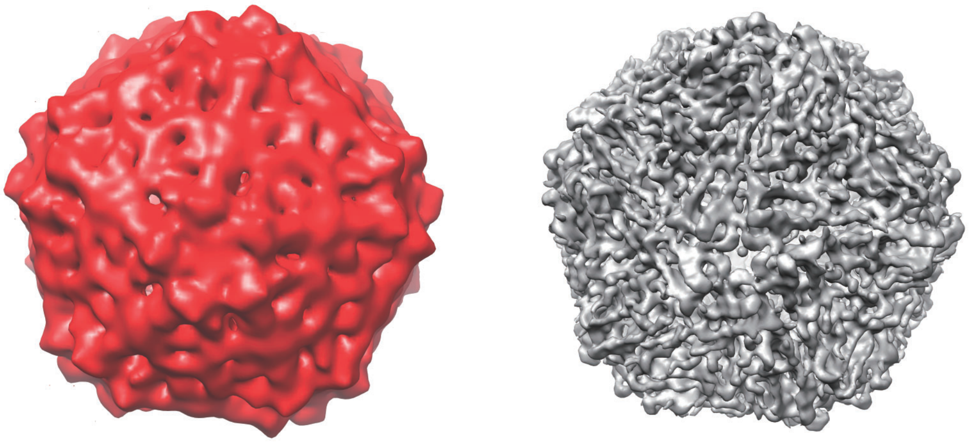

A

3D reconstruction of PCV3 VLP (EMD-6935)

B

Cryo-EM structure of PCV2 VLP (EMD-6746)

Fig S3. Structural comparison of PCV3 with PCV2 structures

Fig S4. Bi et al.

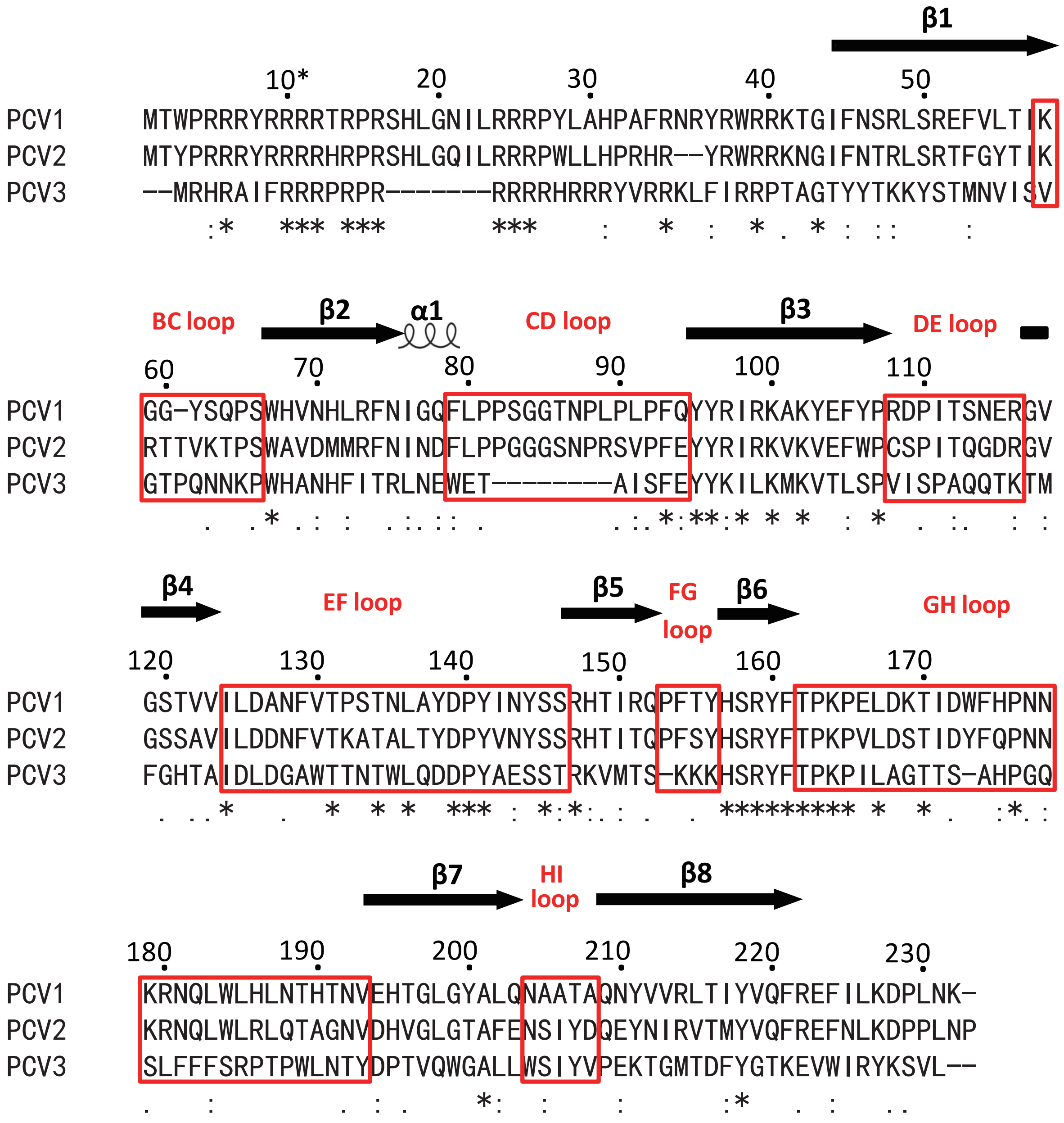

Sequence alignment of PCV1, PCV2 and PCV3 capsid protein.

Fig S4. Multiple sequence alignment of full length PCV1, PCV2 and PCV3 capsid proteins. The capsid protein sequences for three types of porcine circovirus members, PCV1, PCV2 and PCV3 were aligned using ClustalW. The secondary structures (α-helices and β-strands) based on PCV2 are displayed as helices and ribbons above the aligned sequences and the seven exposure loops are depicted and shown in box.

a) (*, asterisk) indicates positions which have a single, fully conserved residue.

b) (:, colon) indicates conservation between groups of strongly similar properties - scoring > 0.5 in the Gonnet PAM 250 matrix.

c) (., period) indicates conservation between groups of weakly similar properties - scoring =< 0.5 in the Gonnet PAM 250 matrix.

Fig.S5 Bi et al.

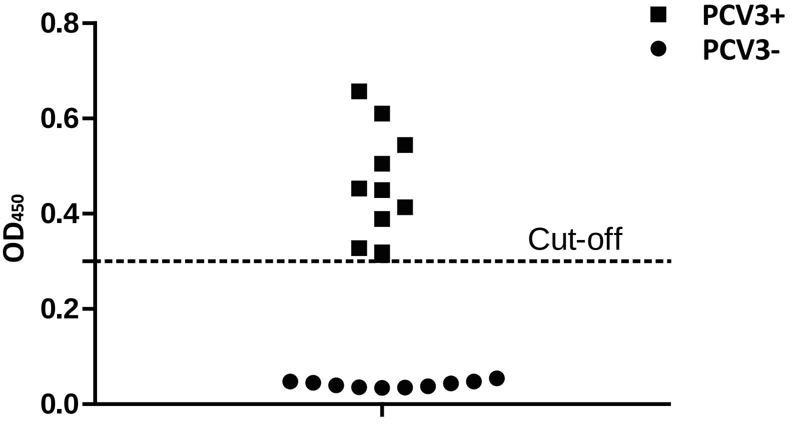

Indirect ELISA for clinical serum samples

Fig S5. Indirect ELISA results for clinical serum sample detection. The PCV3 negative serum samples were indicated by black dots. The PCV3 positive serum samples were indicated by black squares. As shown, 45 the OD_{450} values of PCV3-negative serum samples range from 0.034 to 0.054 with average value of 0.042 measured by established indirect ELISA in this study. By contrast, the OD_{450} values of PCV3-positive serum samples range from 0.311 to 0.657 with average value of 0.464. The differences of OD_{450} values between PCV3-positive and -negative serum samples were significant.

S1 Table. Bi et al.

S1 Table. Details of loops region of PCV2 and PCV3 capsid protein

	PCV2		PCV3	
	Residues	Aligned sequence	Residues	Aligned sequence
BC loop	58-66	KRTTVKTPS	51-59	VGTPQNNKP
CD loop	79-94	FLPPGGGSNPRSVPEE	72-79	WETAISFE
DE loop	108-116	CSPITQGDR	93-101	VISPAQQTK
EF loop	124-146	ILDDNFVTKATALTYDPYVNYSS	109-131	IDLDGAWTTNTWLQDDPYAESST
FG loop	153-156	PFSY	138-140	KKK
GH loop	162-193	TPKPVLDSTIDYFQPNNKRNQ LWLRLQTAGNV	146-176	TPKPILAGTTSAHPGQSLFFF SRPTPWLNTY
HI loop	204-208	NSIYD	187-191	WSIYV
